# Protocol for a quasi-experimental study examining the effect of a ball skills intervention on four domains of preschooler development

**DOI:** 10.1017/S1463423621000645

**Published:** 2021-11-10

**Authors:** Hua Wu, Wichai Eungpinichpong, Hui Ruan, Xinding Zhang, Sansan Wang, Caijin Ding

**Affiliations:** 1 Faculty of Associated Medical Sciences, Khon Kaen University, Khon Kaen, Thailand; 2 Faculty of Physical Education, Hainan Normal University, Haikou, China; 3 Division of Physical Therapy, Faculty of Associated Medical Sciences, BNOJHP Research Center, Khon Kaen University, Khon Kaen, Thailand; 4 Central Kindergarten of Haikou, Haikou, China

**Keywords:** ball skills intervention, emotional competence, executive function, fundamental movement skills, kindergarten, physical fitness

## Abstract

**Background:**

Preschooler inactivity and insufficient motor development have serious long-term consequences. The Chinese Ministry of Education launched a nationwide football-focused pilot project aimed at kindergartens in 2019 and issued the policy “Notice on the Establishment of National Football Kindergartens” in 2020. However, the impact of fundamental movement skills (FMS) interventions on other aspects of child development is unclear.

**Aim:**

This study will evaluate the effects of ball skills physical education projects on the development of Chinese preschoolers’ physical, motor, cognitive, and social competencies and examine the influencing factors.

**Method:**

This is a quasi-experimental study evaluating how well the “Hello Sunshine” curriculum project promotes children’s development over 10 weeks. The trial will be conducted from September 2021 to November 2021 in 12 classes from 3 kindergartens with a total of 249 children aged 4 to 6 years in Haikou, China. Pre- and posttest analyses will include tests on participants’ physical fitness, FMS, cognitive self-regulation, and emotional competence. Participants’ background information will be collected through questionnaires answered by parents and teachers. The intervention will focus on game-based basic ball skills.

**Findings:**

If this intervention provides evidence that these skills improve children’s multidimensional development, it will support the promotion of similar programs in China. We will also outline the social-ecological factors affecting the intervention’s results, providing further information for improving pedagogical methods related to preschool ball skills.

## Introduction

The preschool years form an important stage in the development of children’s physical, motor, socioemotional, and cognitive skills (Chinese Ministry of Education, 2012; Leppänen *et al.*, 2016; Van Capelle *et al.*, 2017; Zeng *et al.*, 2017). From Piaget’s theory, we know that motor skill development in early childhood enables children to perceive the world, gain self-knowledge, and interact with others, which further facilitates improvement in other developmental domains (Goodway *et al.*, 2011). Thelen (1992) also stated that motor skills develop interactively with perceptual, social, and cognitive abilities. However, some evidence indicates that once children enter kindergarten, this interactive relationship seems reduce or become limited to specific domains (Hagmann-von Arx *et al.*, 2016; Kenny *et al.*, 2016; Libertus and Hauf, 2017; Cadoret *et al.*, 2018). While, little is known about the relationship between preschoolers’ motor skills and the development of other critical domains (such as physical fitness, and socioemotional and cognitive skills). Stodden *et al.* (2008) suggested that physical activity and motor skill competence might promote each other in early childhood. Furthermore, some systematic reviews have shown evidence that higher physical activity is associated with improved physical and psychosocial health indicators in preschool (Timmons *et al.*, 2012; Carson *et al.*, 2016, 2017).

Currently, in the area of health care, intervention projects designed to prompt physical activity and prevent obesity in children share three characteristics: 1) preschoolers are targeted; 2) children are provided with structured interventions carried out by teachers and experts, mostly in kindergartens or other child-centric contexts (Logan *et al.*, 2012; Van Capelle *et al.*, 2017; Hesketh *et al.*, 2017); and 3) they focus on fundamental movement skills (FMS) development, especially objective control skill interventions, like the CHAMP project (Robinson *et al.*, 2017; Veldman *et al.*, 2017; Palmer *et al.*, 2019), the Head Start curriculum (Robinson and Goodway, 2009), the SKIP project (Mulvey *et al.*, 2018; Taunton *et al.*, 2018), and other structured skill classes implemented by physical education (PE) teachers (Wadsworth *et al.*, 2020) or non-experts (Palmer *et al.*, 2020).

Learning and developing FMS competence, as supported by theoretical motor development models, is critical in preschool-age children. FMS is considered the building block of more complex and advanced skills, which means that mastering these abilities makes it easier for children to learn more specialized skills later on (Seefeldt *et al.*, 1980; Clark and Metcalf, 2002; Gallahue and Donnelly, 2007).

FMS terms such as “motor performance,” “motor proficiency,” “motor ability,” and “motor coordination” (Cattuzzo *et al.*, 2017) are often used interchangeably and inconsistently within the literature. In our study, we used the FMS definition: “basic learned movement patterns that do not occur naturally and are suggested to be foundational for more complex physical and sporting activities” (Barnett, Stodden, *et al.*, 2016). Thus, FMS includes locomotor (e.g., run, hop, slide), object control (e.g., dribble, throw, kick, also known as manipulative or ball skills) (Okely *et al.*, 2001), and stability skills (e.g., beam walk and one-foot balance) (Goodway *et al.*, 2011).

Evidence suggests that FMS has a positive association with multiple aspects of health in children, such as their physical activity levels, cardiorespiratory fitness, muscular strength and endurance, and healthy weight maintenance (Stodden *et al.*, 2008; Lubans *et al.*, 2010; Robinson *et al.*, 2015; Cattuzzo *et al.*, 2017), which are the foundations for the health and well-being of children. Studies have explored the association between FMS and variables of children in kindergartens, namely, social-emotional behaviors (Piek *et al.*, 2008; Robinson *et al.*, 2016; Ma *et al.*, 2019) and cognitive and academic performance (Planinsec, 2002; Niederer *et al.*, 2011; van der Fels *et al.*, 2015; Fang *et al.*, 2017; Cadoret *et al.*, 2018; Michel *et al.*, 2019).

In most countries, however, FMS competence is insufficiently developed in preschoolers (Taggart and Keegan, 1997; Hardy *et al.*, 2010; Robinson *et al.*, 2017), with this deficit continuing into primary school (van Beurden *et al.*, 2002; Hardy *et al.*, 2012; Spessato *et al.*, 2013; Mukherjee *et al.*, 2017; Wu *et al.*, 2017). Recently, a systematic review (Bolger *et al.*, 2020) of studies in typically developing children (3–10 years) worldwide demonstrated that FMS levels were “below average” to “average” according to the Test of Gross Motor Development-2 and that ball skills proficiency was less than locomotor skill. It was, therefore, recommended that in order to address these limitations, FMS interventions should be taught, practiced, and reinforced to children (Robinson and Goodway, 2009).

To date, multiple intervention projects have been implemented, but their effectiveness in healthy preschoolers remains controversial. There are many issues around this topic. First, some interventions lack a rigorous design and only record the outcomes directly after the intervention, with a lack of medium- and long-term follow-up (Wick *et al.*, 2017). Second, some interventions have limited assessment variables and focus narrowly either on a certain aspect of children’s health or risk development. Third, differential outcomes are often ignored in intragroup cases. Finally, there is a general lack of exploration of the social-ecological factors correlating with FMS competence (Barnett *et al*., 2016). It can be argued that FMS intervention projects should evaluate the interconnection of motor skill development with physical fitness, cognitive, and social abilities (subject to physical and environmental constraints), as these are the main aspects determining preschoolers’ health (Westendorp *et al.*, 2014; Leonard, 2016; True *et al.*, 2017; Zeng *et al.*, 2019).

### Background

In recent years, the Chinese government has promulgated policies to promote the development of basketball (Hu fengshen, 2017) and football (Ministry of Education of the People’s Republic of China, 2019) in kindergartens in order to develop students’ physique and talents. Although there is a rise in sport-centric characteristics among kindergarteners, and some successful examples, the question of how to use ball activities to promote early childhood development still needs exploration, along with a scientifically comprehensive evaluation of children’s developmental benefits resulting from participation in these games.

Based on the current cultural context and the potential importance of children’s ball skills for the long-term promotion and maintenance of health-related fitness (Stodden *et al.*, 2014), our research will use ball activities to promote FMS, cognitive performance, social skills development, and other health benefits. The “Hello Sunshine” (HS) project is a PE intervention project for preschoolers that uses various ball activities (football, basketball, badminton, table tennis, baseball, golf, handball, bowling, and snooker) as a strategy to promote multiple aspects of children‘s development. The HS project not only teaches football and basketball but also guides children to learn and experience different ball skills and have more fun with a variety of tasks and environmental constraints. It is likely that not every child will enjoy ball games and this gives us an opportunity to explore how social-ecological factors may influence intervention outcomes. It is hoped that this study will provide a basis for a pedagogical method to improve preschooler’s ball skills.

### Theoretical framework

**Figure 1.**
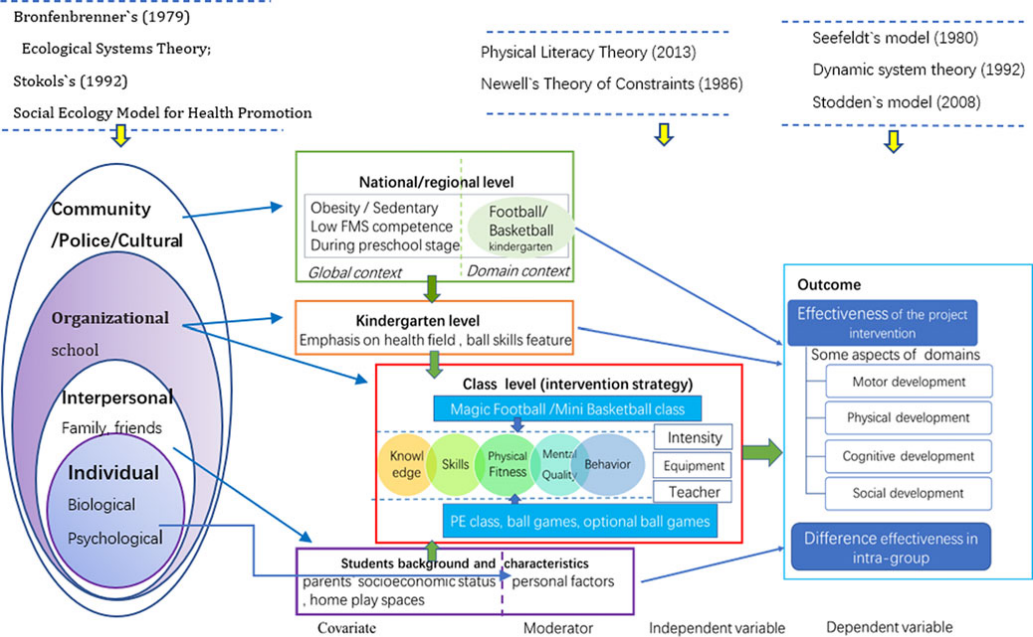
Theoretical framework of this project

It is well known that theories and models used in the health promotion and educational fields provide a framework for developing, understanding, and carrying out interventions (Glanz *et al.*, 2008). Our theoretical framework, including its concepts, models, and theories, is described below (Figure 1).

Our theoretical framework utilized Bronfenbrenner’s ecological systems theory (Bronfenbrenner, 1979) and Stokols’ social ecology model for health promotion (Stokols, 1992) to guide the targeting of specific factors when designing this intervention project and explaining its outcomes. According to the proposed model, children’s behavior is influenced by the individual, their affiliations to people, organizations, and the community, and at the public policy level, and these are collectively known as social-ecological factors. Cool *et al.* (2011) identified that parental education, father’s physical activity, and parental rearing pattern had a relationship with preschooler’s FMS performance. In a meta-analysis, Barnett *et al*. (2016) found that FMS competence was correlated with individual factors, such as age, healthy, weight, range, sex (male), and family socioeconomic status (higher). Zeng *et al.* (2019) suggested the different components of a preschooler’s FMS have multidimensional relationships with social-ecological factors at the child, family, and environmental levels, and Niemistö *et al.* (2020) considered individual factors (age, physical, and specific temperament) to be the most important predictors of FMS.

Teachers and teaching strategies at the kindergarten level are hypothesized to be important factors when initiating children’s FMS programs. At this point, multiple human–environment interactions take place, which, in addition to children’s characteristics, influence their FMS levels and the effects of interventions.

Our intervention project will be based on the concept of cultivating physically literate individuals. Physical literacy was first proposed by Whitehead (2013) and has now become globally accepted as a key element in quality PE, according to the United Nations Educational, Cultural, and Scientific Organization (McLennan and Thompson, 2015). The ball skills teaching method is game-based and pursues the interaction of different constraints – task, environment, and performer (Newell 1986).

The outcomes of this project will allow us to explore the effects of this intervention on the physical, motor, cognitive, and social development of preschoolers, according to the motor development process and dynamic systems theory (Thelen, 1992), as well as the relationship among physical activity, FMS perceived competence, and health-related fitness as Stodden’s model (Stodden *et al.*, 2008). Additionally, based on existing studies, we used the social-ecological model to explain individual differences in the intervention’s outcomes (Glanz *et al.*, 2008; Bellows *et al.*, 2013; Taunton *et al.*, 2018; Zeng *et al.*, 2019).

## Materials and methods

The study protocol complies with the guidelines of the SPIRIT 2013 statement (Chan *et al.*, 2013). The trial was registered with the Chinese Clinical Trial Registry (registration number: ChiCTR2000035414) on 10 August 2020 and will be carried out in accordance with the Declaration of Helsinki.

Children, aged 4–6 years, from three predetermined kindergartens will participate. The three kindergartens are categorized based on three different PE programs provided for the children: a Hello Sunshine (HS), an ordinary PE (OPE), and a free play (FP) depending on their existing PE curriculum.

### Aim

This study aims to conduct a “Hello Sunshine” curriculum project and evaluate its effects on the development of physical, motor, cognitive, and social competence in preschoolers. It also aims to determine the social-ecological factors influencing the intervention’s effect.

### Objectives

- To assess the effects of the HS project on the development of children’s physical (anthropometric, physical fitness), motor (FMS), cognitive (perception, self-regulation), and social (emotional comprehension) development and to contrast intervention effects between different PE course groups.
- To use a social-ecological perspective to explore the factors affecting different intragroup outcomes in the intervention groups.
- To identify the main factors impacting the efficiency of the HS intervention at an intragroup level.

### Research questions

The following research questions are proposed in this study:

1. What are the effects of the HS project interventions on the physical fitness and motor development of preschool children, as well as on their cognitive and social competencies?
2. How do intervention effects differ among the HS group, the OPE group, and the FP group?
3. To what extent are the intervention gains on the HS groups mediated by personal factors?
4. What are the main social-ecological variables related to differences in the intervention outcomes?
5. To what extent are the outcome variables related to each other?

### Hypotheses

Based on these five research questions, we hypothesize that children in all groups will exhibit improvements in developmental outcomes across the 10-week period. We predict participants in the HS groups will report significantly higher scores on physical fitness, FMS, and cognitive self-regulation tests at follow-up, compared to baseline. We also expect improvements in these indicators to be significantly greater when compared to the alternative programs (OPE and FP).We also hypothesize that differential intervention effects within the HS groups will be associated with the individual, family, and environmental factors.

### Methods and design

#### Study design

A quasi-experimental design study will be employed.

At the beginning of the September 2021 school semester, preparations and baseline data collection will be carried out over 2 weeks, with an intervention period running for 10 weeks. This will be followed by a posttest period and a 6-week follow-up. At the beginning of the February 2022 semester, the third round of testing will be conducted. Given the uncertainty of the pandemic, if the school temporarily closes again, the project will be postponed until the next semester or the next opportune time.

**Figure 2.**
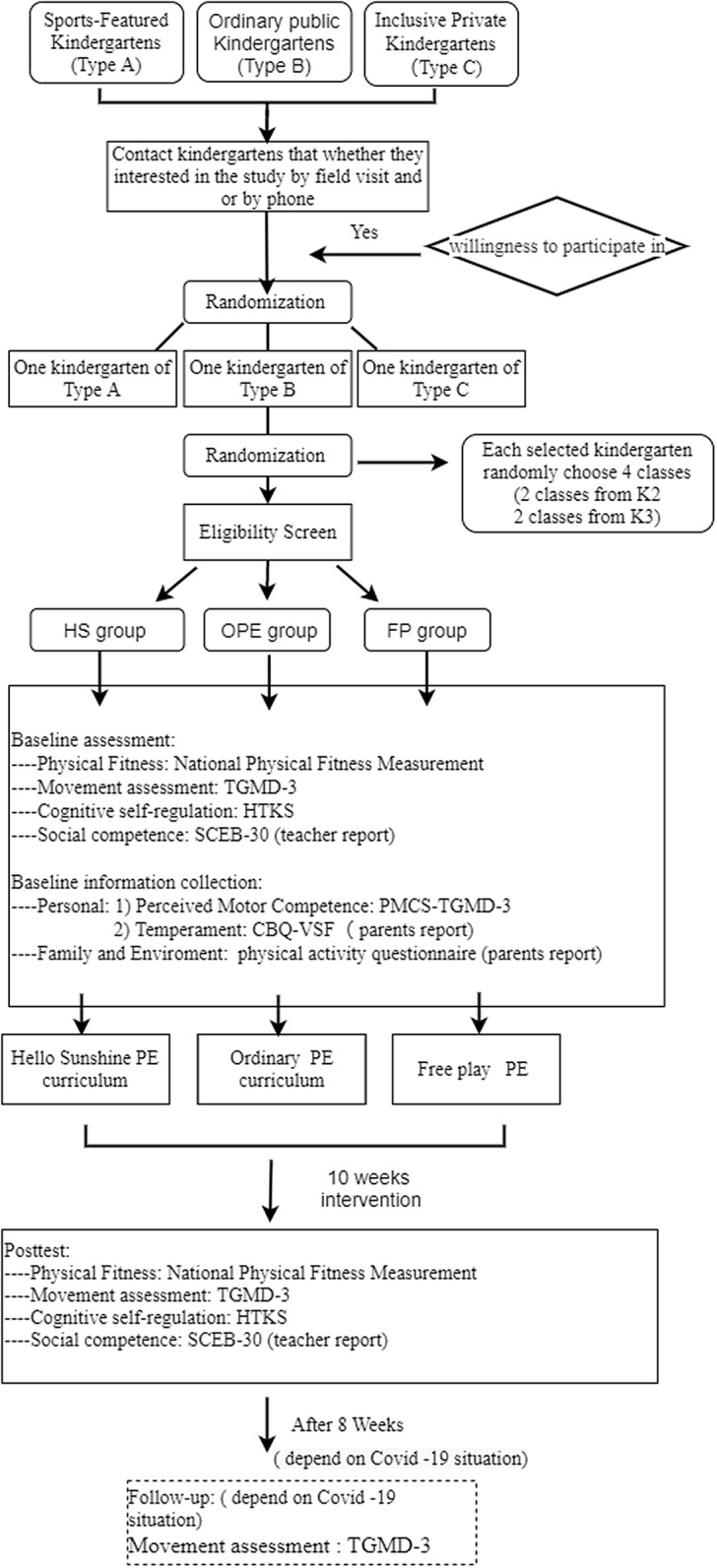
Study procedure. HS: Hello Sunshine PE curriculum; OPE: Ordinary PE curriculum; FP: Free play PE

This study’s procedures are illustrated in Figure 2.

## Participants

The study will be conducted in three types of kindergartens in the center of Haikou city, China. Sport-featured kindergartens, public kindergartens, and inclusive private kindergartens in the same district will be assigned. Two classes in K2–K3 grade (children aged 4–6 years) will be randomly selected from these kindergartens and will be allocated as HS group (from sport-featured kindergarten), OPE group (from public kindergarten), and FP group (from inclusive private kindergarten).

The inclusion criteria are: 1) healthy and typically developing children willing to participate, 2) aged 4–6 years, 3) with the informed consent of their primary caregivers, 4) the primary caregiver must have a smartphone to answer the questionnaire. Children 1) who have difficulty in communication, unexplained health problem, 2) who have a contraindication to exercise such as congenital heart disease, asthma, fever, malaise, diarrhea, and 3) who have additional training from a sports club will be excluded.

## Sample size determination

We used WinPepi software to calculate the sample size with reference to Westendop’s research (2014) while ensuring it would be larger than those used in other comparative studies (Robinson *et al.*, 2017; Taunton, Mulvey and Brian, 2018). The following assumptions were used: significance level of 5%; power of 80%; and confidence level of 95%, inflated to compensate for a 5% non-inclusion rate. As a result, at least 249 children (83 in the HS group, 83 in the OPE group, and 83 in the FP group) will participate in this study.

## Randomization

The children aged 4–6 years within four classes (two classes selected from K2 and K3 grade, separately) will be randomized from the designated kindergartens using Research Randomizer software. The preschoolers in each class who meet the eligibility criteria will be screened by the kindergarten teachers and staff. To avoid changes in teachers and classmates affecting the results of this course, we will intervene in the form of a nature class. In total, 12 nature classes in grades K2 and K3 from the 3 kindergartens will be assigned to a PE curriculum in their respective schools, that is, 4 classes in the sport-featured kindergarten with “Hello sunshine” PE curriculum, the OPE group will have ordinary PE classes, the PE class in the FP group will be free play. This sequence will then be concealed in an opaque envelope.

## Blinding

Trained graduate students and senior undergraduate students will act as researcher. The researcher will not know which children will have had which intervention until the end of the study. However, each of the participants will know which group he/she is belongs to. Thus, only single blind (blind the researcher) will be applied in this study. The data will be renamed and converted into statistics by the second author.

### Interventions

In the study, the participants will be divided into three groups. The PE teacher will use various kinds of ball game-based curriculum in the HS group. The OPE group will have the ordinary preschool PE class that uses the kindergarten-based syllabus with a PE teacher (ball skills are not the main teaching contents). The FP group will play freely in outdoor activities with no guidance from teacher. The three groups will have the same daily life at kindergarten, except for the content of the PE class and outdoor time. PE classes will be 25 or 30 min, one or two times per week (depending on the student grade). Outdoor time will be implemented for 30 min per day, 5 days a week for 10 weeks during the intervention period. All three methods (HS, PE, and FP) are believed to improve motor skills and physical activity. The differences in the three methods are described below. This description follows the Template for Intervention Description and Replication checklist and guidelines for better reporting of interventions (Hoffmann *et al.*, 2014).

#### HS group

The HS project’s design is based on the physical literacy framework (Whitehead, 2013). It is a well-structured, age-demarcated, PE curriculum that includes five modules: knowledge, motor skills, physical quality, psychological quality, and health behavior. Each module has different course objectives and content in accordance with age. The HS curriculum consists mainly of ball skills activities including teaching or practicing ball skills in PE class, ball club time, and autonomous regional area in outdoor time. It consists of nine kinds of ball activities, such as football, basketball, badminton, table tennis, baseball, golf, handball, bowling, and snooker. K2 students will have a PE class (one ball topic for 25 min) and a ball clubs’ class (children volunteer to join any club for 60 min) once a week. In addition, they will have one football activity (autonomous regional play) in the afternoon outdoor time, daily. K3 children will have PE class (football or basketball) twice a week and ball club activities once a week, and football topic activity two times and golf topic activity in outdoor activity time once. In the PE class, ball game-related general knowledge, ball skills, and so on will be taught. In ball clubs’ time, children will participate and practice in ball games with the PE teacher. During outdoor activity, children will play in an autonomous regional ball area with supervision by the vice-class teacher for 30min.

An experienced PE teacher will teach basic ball skills in the context of a game or story and motivate the children to complete the task, while also enjoying themselves. The teacher will guide the children through the preset game scenarios, while demonstrating and explaining basic ball skills and their task or activity execution via prompt words that remain consistent with the teaching plan. Timely encouragement and feedback will also be provided to the children. During the outdoor class, high-autonomy interventions, wherein the children have the freedom to self-navigate through a ball skills game station are to be conducted.

Taking K3 for example, size 3 footballs or size 4 basketballs will be used during the nature classes to help the children learn and practice in the PE class. Hoops, traffic cones, disk cones, inclined plates, and mini-goals will be used as teaching aids. Participants will undergo 30-min classes, including jogging and playing games for a warm-up of 5–8 min, followed by 20 min of learning ball skills (PE class) or playing ball activities (outdoor times), and finally, 2–5 min of cooldown (using the ball to stretch and lightly pat themselves along with music). The teachers will monitor the intensity of play by observation of facial expression and color, and respiratory rate as recommended by the physical activity guidelines for Chinese preschool (Working Group on Physical Activity Guideline for Chinese Preschoolers Aged 3–6 Years, 2020) and assist children to engage in energetic play (NHS GGC, 2017; WHO Team, 2019).

#### OPE group

Children will have PE class with the PE teacher according to the assigned kindergarten textbook (Hainan edition) for monthly theme teaching, not focused on ball skills. K2 and K3 will have one PE class in the afternoon, five times/week and the same outdoor time as the HS group, but structured as FP.

#### FP group (control group)

The kindergarten will have a space for children to play freely with all kinds of sports equipment (slide, sand, climbing frame, etc.) They will be supervised by vice-class teachers without PE backgrounds.

## Fidelity

The fidelity measurements of the HS and OPE interventions are based on the curriculum lesson plan and curriculum summary provided by kindergarten teachers, which record the theme, goal, design, and other contents of the course. Research group experts will attend one class per week in each kindergarten at random to check that reporting from teachers accurately reflects the activities delivered.

### Outcome measures

The assessment process will use a variety of methodologies (scales, field tests, questionnaires, and ability-based tasks) and multiple informants (teachers and parents). An overview of the measurement process is shown in Table 1.

**Table 1.** Domains and factors measured by the tests

Domain	measure	Items	Tools
Primary outcome			
Physical development	Anthropometrics	Hight Weight	Hight and weight scale
	Physical fitness	Strength Balance Agility Flexibility	Tennis throwing test Standing long jump Walking on a balance beam 10-m SRT Sit and reach
Motor development	Movement assessment	FMS	TGMD-3
Cognitive development	Cognitive self-regulation	Cognitive self-regulation	HTKS
Social development	Social competence	Emotional competence	SCEB-30 (teacher)
Secondary outcome			
Personal	Basic information	Perceived motor competence	PMCS-TGMD-3
		Temperament	CBQ-VSF (parents)
Family environment	Basic information	Parents’ age Parents’ education Physical activity and home play spaces	Questionnaire (parental report)
	Basic information	Sports injure Attendance rate	Recorded by teacher
Epidemiology	Recorded information		Recorded by school doctor

#### Anthropometric measures

The height and weight of the children will be measured in the morning by the research assistants. The children will wear light clothing, without shoes, and their standing height and body weight will then be measured using an electronic scale. Body mass index will be calculated as weight/height squared (kg/m^2^), and the weight status will be classified by Chinese preschooler overweight and obesity criteria (Li *et al.*, 2010).

#### Physical fitness measures

We will choose items from the National Physical Fitness Measurement Standards Manual (State Sport General Administration, 2003). Tennis ball throwing, sitting, and reaching tasks represent aspects of health-related fitness (muscle strength, flexibility) and standing long jump, walking on a balance beam, and the 10-m SRT test represent skill-related physical fitness (explosive strength, balance, and agility).

#### Movement measures

##### Gross motor skills

FMS will be assessed using Dr Ulrich’s Test of Gross Motor Development-3 (TGMD-3) (2019), a validated and reliable assessment tool for measuring gross motor skill competence in children aged 3–10 years (Dale A, Ulrich, 2013; Brian *et al.*, 2018). The TGMD-3 is subdivided into two subscales: locomotor (running, galloping, 1-legged hopping, skipping, jumping, and sliding) and ball skills (2-hand striking, 1-hand striking, dribbling, kicking, catching, overhand throw, and underhand throw). Each skill is evaluated based on three to five performance criteria. If a child demonstrates the correct performance criteria, a score of 1 for each criterion is given; otherwise, they receive a score of 0. Children complete one practice and then two formal trials. Coding is only completed for the two formal trials, which are summed to calculate the raw score.

#### Cognition measures

##### Cognitive self-regulation

Executive function (EF) is used to describe “higher or meta” cognitive function (Miyake *et al.*, 2000) and is critically important in the overall neuropsychological functioning of the developing child (Isquith *et al.*, 2005). The Head–Toes–Knees–Shoulders (HTKS) test (Ponitz *et al.*, 2009; Meador *et al.*, 2013) assesses children’s behavioral self-regulation, which reflects their ability to transform executive functioning into explicit behaviors (Becker *et al.*, 2014), including a measure of inhibitory control, working memory, and attention focusing (Ponitz *et al.*, 2008). The test has strong interrater reliability and is a good evaluator of the behavioral self-regulation of children aged 4–8 years from different cultural backgrounds (Ponitz *et al.*, 2008). The test is divided into two parts. The first task is Head to Toes (HTT) and the child is required to react in the opposite way to two oral instructions from the examiner (when the child hears “touch your head” they should respond by touching their toes, and vice versa). The child will perform six practice trials with feedback followed by 10 test trials. If the child passes the HTT section, they will complete an advanced task, which mixes the knee and shoulder commands. In this task, the child should respond to the new commands (“touch your shoulders” and “touch your knees”) by performing the opposite action and touch their knees or touch their shoulders, respectively. This section concludes with 10 test trials that mix touch your head or shoulders, knees or toes oral prompts. HTKS is composed of 26 irregular instructions (16 trials of HTT and 10 trials of HTKS). Children get 2 points for correct responses, 0 points for incorrect responses, and if they immediately correct a wrong action, they score 1 point. Thus, the score range is 0 to 52 points (Meador *et al.*, 2013). This test was previously used in the study of Chinese preschool children (Ma *et al.*, 2019).

## Social competence

Emotional ability is crucial to building interpersonal relationships (Parke, 1994). Even during the preschool period, emotional competence contributes to social competency and has long-term implications (Denham *et al.*, 2003).

## Emotional competence measure

The shortened version of the Social Competence and Behavior Evaluation Scale (SCEB-30) (LaFreniere and Dumas, 1996) will be used for measuring participants’ emotional competency levels and will be utilized in each class with the classroom teacher. The test consists of three subscales: social competence, anger aggression, and anxious-withdrawal. It has high interrater and test–retest reliability, internal consistency, and over 6-month temporal stability (LaFreniere and Dumas, 1996). The Chinese revised version has proven to have good reliability and validity when applied for Chinese preschoolers (Liu *et al.*, 2012).

### Covariate and mediator

#### Personal factors

Age will be calculated as the children’s month-based ages, as it is going to affect the intervention’s outcome. Participants’ perceived motor competence will be measured with a pictorial scale for perceived movement skill competence (PMSC) that builds upon the TGMD-2 (Barnett *et al.*, 2015). It consists of 13 pictures illustrating cartoon boys (boy version) or girls (girl version) performing TGMD-3 skills. It shows middle to high instrument reliability and face validity in Chinese children (Diao *et al.*, 2018). Children will be shown illustrations by the examiner and judge whether the cartoon character is good at doing the skills, then choose between four Likert scale options (1 to 4, from “not too good” to “really good”).

Individual temperaments are expected to be predictive of the differential effects of this motor skills intervention (Taunton *et al.*, 2018), and they will be assessed using the 36-item Children’s Behavioral Questionnaire-Very Short Form (CBQ-VSF) (Putnam and Rothbart 2006). The test is divided into three broad dimensions: surgency, negative affect, and effortful control and it demonstrates acceptable internal consistency. It is a very short form based on the well-established parent report measure of temperament CBQ questionnaire for children aged 3 to 8 years (Rothbart *et al.*, 2001) and it has medium to high reliability and validity confirmed by studies in multiple countries. The CBQ-VSF provides moderate reliability of scale scores for assessing temperamental characteristics in preschoolers (de la Osa *et al.*, 2014).

#### Family factors

Parents of the participants will fill out questionnaires to provide additional information about their sociocultural characteristics. For example, occupational status will be defined according to the following categories ranging from low to high: unemployed, worker, education and teaching, self-employed, management, executive position, and professional (Vandendriessche *et al.*, 2012) and education level will be classified from low to high level: 1) secondary school or less, 2) trade certificate/diploma, and 3) university qualification (Hacke *et al.*, 2019). Family income will not be asked for privacy reasons.

#### Environment factors

##### Parental report of physical activity

Children’s physical activities promote the development of their FMS (Fisher *et al.*, 2005; Stodden *et al.*, 2008). Parental reports of their preschool-aged children’s physical activity levels are feasible in studying the home (available recreational space inside the home) and environment (community) of population-based samples (Burdette *et al.*, 2004).Our survey will explore this factor using self-designed parent reports of physical activity based on Palou’s (2019) study. The parents will be asked to answer the following questions:

1. “Your children’s physical activity time every day after school is …?” The response options are 1 = 30 min or less, 2 = between 30 min and 1 h, 3 = between 1 h and 2 h, 4 = between 2 h and 3 h, 5 = more than 3 h.
2. “Your children’s screen time after school is …? The options are: 1 = less than 30 min, 2 = between 30 min and 1 h, 3 = between 1 h and 2 h, 4 = between 2 h and 3 h, 5 = more than 3 h. (WHO Team, 2019; Working Group on Physical Activity Guideline for Chinese Preschoolers Aged 3–6 Years, 2020).
3. “How many times do you perform moderate or vigorous exercise for at least 30 min per week ….?” Optional answers are: 1 = 0 times, 2 = 1 to 2 times, 3 = 3 to 4 times, 4 = 5–6 times, 5 = more than 6 times (Li *et al.*, 2020; WHO 2020).
4. “How much space in your house for child recreational play?” The size options are: 1= < 20 m^2^, 2 = 20 to 50 m^2^, and 3 = > 50m^2^ (Bardid *et al.*, 2013).
5. “How often is your child allowed to play in the community playground or sports facilities ?” Optional answers are 0 = never, 1 = during weekends, 2 = every now, and 3 = nearly daily (Niemistö *et al.*, 2020).

### Study procedure

After conducting the preliminary research work, the researchers will contact three kinds of kindergartens in this district and communicate with the directors to enquire whether they are interested in research on the health benefits of physical activity in young children. It was agreed that once the study has been approved by the ethics committee, the researchers will recruit participants from selected kindergartens. The kindergartens will send invitations to parents, along with information about the project and an informed consent form. After the collection of the signed consent forms, eligible participants will be screened by the kindergarten teachers, and the researchers. The baseline measurements will be conducted in the initial 2 weeks and all the participants will experience the same assessment at the end of the 10 weeks intervention. The experimental process will be administered as shown in the flow diagram (Figure 2). The physical fitness test, TGMD-3 test, and psychological test (PMCS and HTKS) tasks will be tested in random order in each kindergarten and based on their schedules and space availabilities both at baseline and after 10 weeks intervention, should finish within 4 days in each kindergarten with trained research students responsible for grouping the test items. All children will be encouraged with snacks or stickers to complete all tests. Video recordings will be used for later scoring and checking. The participants will be given a separate and quiet space to take the psychological test. Parents will receive their digital-based questionnaires at a parents’ meeting. They will fill these out and submit them on the day. The head teacher of each class will fill in the SCEB-30 questionnaire in 1 week.

## Results

Data will be imported from Excel 2019 to IBM SPSS version 26.0 for inferential statistical analysis. The normal distribution of the data will be checked before determining the descriptive statistics for all variables (means and standard deviations). The participants’ basic information will be descriptive, including age, gender, and BMI. The primary outcomes will be expressed as continuous. If data are approximately normal distribution, the effect of the PE curriculum intervention on dependent variable (FMS, or TGMD, or HTKS, or SCEB score) will be investigated with repeated ANOVAs within factor: time (pre and post), between factors: group (HS, OPE, and FP) and gender (boy and female) and Bonferroni post hoc tests. Further, the relationship between FMS and other domains will be identified by regression analysis. Finally, the potential influence of individual factors (such as age, gender, and temperament) on the intervention effect will be investigated by comparing the gain in FMS score and subscale. Multiple regression equation is used to further explore the extent to which social-ecological factors contribute to the FMS score level at baseline. Statistical significance will be set at *P* < 0.05.

## Discussion

There is currently a diverse collection of interventions for improving PA and FMS in young children, but a dose–response relationship and consequent intervention effects are uncertain and incomplete. For example, some studies have outlined immediate effects (without follow-up), showing a significant increase in PA (Puder *et al.*, 2011; Gao *et al.*, 2019) and FMS (Wick *et al.*, 2017) in intervention groups. Other studies indicate no significant changes in either PA (Cliff *et al.*, 2011; Palmer *et al.*, 2019) or FMS (Bonvin *et al.*, 2013). Hence, any causal relationship between PA, FMS (Van Capelle *et al.*, 2017), and perceived motor skills in preschool children remains unclear (Stodden *et al.*, 2008). Moreover, few studies have focused on the cognitive, social, behavioral, and other developmental changes of preschoolers and the influence of certain covariates (e.g., social-ecological factors) on the maximization of the intervention’s effect.

Although football and basketball classes tend to be popular in Chinese kindergartens, there is a lack of systematic scientific research on their effects on children’s overall health. This study is the first quasi-experimental study to explore three kinds of PE education in terms of their effects on the physical, motor, cognitive, and social development of preschoolers. The purpose of this project is to explore whether these ball game interventions have differential influences on the domains of children’s development, compared to a control condition (traditional PE class). It will further explore the influence of children’s social-ecological backgrounds and the intragroup effects on the intervention’s results.

## Conclusions

Physical inactivity and low FMS competence in preschool children is a global challenge. This project has been designed to be implemented within a realistic context with a policy-advocating aim, to explore the effectiveness of improving ball skills on children’s multidimensional development. Our findings will also attempt to explain the problems in this intervention and provide evidence for the development of effective PE for children.

### Strengths and limitations

One of the strengths of this project is that the quasi-experimental study comparing three parallel groups of PE in kindergartens using blinded assessors. This study design is implemented in realistic environment where the kindergartens are likely to be more cooperative as they can keep their existing curriculum arrangements. The HS group is mainly focused on ball skills, the other two teaching methods represent the mainstream PE activities employed in most kindergartens; the OPE group covers multiple types of physical activity taught by PE teachers, and the control group’s main characteristic is FP. Our research will involve a comprehensive quantitative analysis of the immediate and follow-up effects of the interventions on four domains of children’s development and will collect information from multiple informants including from children, their parents, and their teachers. Finally, the impact of social-ecological factors on the interventions’ effects will be analyzed.

A limitation of this research will be the large number of items tested in this project, as these will take more time to collect and may stress the children’s patience levels. The short forms of each of the established tests have been selected. Exercise interventions in outdoor environments are also affected by weather conditions, including rain, and/or overheating during high temperatures, and the effects of the COVID-19 pandemic. Such factors may affect both delivery and compliance. Further randomized controlled trial studies conducted in more kindergartens will broaden our understanding of the impact of ball skills learning on children’s development, and a cohort study will be established to explore the long-term effects of ball skill learning on children.
